# Evidence for a Pro-Inflammatory State of Macrophages from Non-Obese Type-2 Diabetic Goto-Kakizaki Rats

**DOI:** 10.3390/ijms251910240

**Published:** 2024-09-24

**Authors:** Amanda Santos de Almeida Silveira, Amara Cassandra dos Anjos Alves, Gabriela Mandú Gimenes, Patrícia da Silva Quessada, Tiago Bertola Lobato, Beatriz Belmiro Dias, Ana Carolina Gomes Pereira, Patrícia Nancy Iser-Bem, Joice Naiara Bertaglia Pereira, Elaine Hatanaka, Laureane Nunes Masi, Tânia Cristina Pithon-Curi, Vânia Gomes de Moura Mattaraia, Sandro Massao Hirabara, Amanda Rabello Crisma, Renata Gorjão, Rui Curi

**Affiliations:** 1 Interdisciplinary Post-Graduate Program in Health Sciences, Cruzeiro do Sul University, São Paulo 01506-000, SP, Braziltiagobertola@hotmail.com (T.B.L.); sandro.hirabara@cruzeirodosul.edu.br (S.M.H.);; 2 Butantan Institute, São Paulo 05585-000, SP, Brazil; 3 Multicenter Graduate Program in Physiological Sciences, Department of Physiological Sciences, Center of Biological Sciences, Federal University of Santa Catarina, Florianopolis, SC 88037-000, Brazil; 4 Department of Clinical Analysis, Federal University of Paraná, Curitiba 80210-170, PR, Brazil

**Keywords:** type 2 diabetes mellitus, insulin resistance, inflammation, GK, GM-CSF, M1, M2, TNF-α

## Abstract

Obesity causes insulin resistance (IR) through systemic low-grade inflammation and can lead to type 2 diabetes mellitus (T2DM). However, the mechanisms that cause IR and T2DM in non-obese individuals are unclear. The Goto-Kakizaki (GK) rat develops IR spontaneously and is a model of non-obese T2DM. These rats exhibit hyperglycemia beginning at weaning and exhibit lower body mass than control Wistar rats. Herein, we tested the hypothesis that macrophages of GK rats are permanently in a pro-inflammatory state, which may be associated with a systemic inflammation condition that mimics the pathogenesis of obesity-induced T2DM. Using eighteen-week-old GK and control Wistar rats, we investigated the proportions of M1 (pro-inflammatory) and M2 (anti-inflammatory) macrophages isolated from the peritoneal cavity. Additionally, the production of inflammatory cytokines and reactive oxygen species (ROS) in cultured macrophages under basal and stimulated conditions was assessed. It was found that phorbol myristate acetate (PMA) stimulation increased GK rat macrophage ROS production 90-fold compared to basal levels. This response was also three times more pronounced than in control cells (36-fold). The production of pro-inflammatory cytokines, such as tumor necrosis factor-alpha (TNF-α), tended to be upregulated in cultured macrophages from GK rats under basal conditions. Macrophages from GK rats produced 1.6 times more granulocyte-macrophage colony-stimulating factor (GM-CSF), 1.5 times more monocyte chemoattractant protein-1 (MCP-1) and 3.3 times more TNF-α than control cells when stimulated with lipopolysaccharide (LPS) (*p* = 0.0033; *p* = 0.049; *p* = 0.002, respectively). Moreover, compared to control cells, GK rats had 60% more M1 (*p* = 0.0008) and 23% less M2 (*p* = 0.038) macrophages. This study is the first to report macrophage inflammatory reprogramming towards a pro-inflammatory state in GK rats.

## 1. Introduction

In 2021, diabetes mellitus (DM) was diagnosed in 537 million people, corresponding to 10.5% of the world’s adult population [1]. Type 2 DM (T2DM) accounts for over 90% of these cases and results from insulin resistance (IR) in peripheral tissues or decreased insulin secretion due to dysfunction in pancreatic β-cells [1]. Additionally, T2DM has been associated with increased plasma free fatty acid (FFA) concentrations, a common characteristic of obesity. The FFAs promote IR by activating protein kinases such as IκB-β (IKKβ), which subsequently phosphorylate insulin receptor substrates (IRS, especially IRS1 and IRS2) in serine residues, instead of tyrosines [2,3,4,5,6]. The activation of IKKβ also promotes the expression of pro-inflammatory genes, such as tumor necrosis factor-alpha (TNF-α) and interleukins (ILs) IL-6 and IL-1β, which activate protein kinases (IKKβ, JNK1 or MAPK8, and p38 MAPK) and cytokine signaling suppressors (SOCS), consequently inhibiting the insulin signaling cascade [2,3,4,7]. Furthermore, the large number of macrophages that infiltrate the white adipose tissue (WAT) of obese individuals contributes to increased plasma levels of pro-inflammatory cytokines [8,9].

However, besides obesity, other factors, including mitochondrial dysfunction, endoplasmic reticulum stress, oxidative and nitrative stress, aging, and systemic inflammation conditions, can lead to IR and, ultimately, the development of TD2M [2,3,10,11]. In Japan, 60% of individuals with T2DM have a body mass index (BMI) lower than 25 kg/m [2,12,13]. Moreover, Chan et al. (2009) [14] associated the predisposition of non-obese Japanese to T2DM with high abdominal and visceral adiposity and lower muscle mass compared to the Western population.

To study the onset and development of T2DM without the complicating factors associated with obesity, the Goto-Kakizaki (GK) rat model was utilized. This spontaneous model of non-obese T2DM was obtained by breeding glucose-intolerant Wistar rats [15]. Previous studies have shown that GK rats have about 50% fewer pancreatic β cells and exhibit glucose intolerance and hyperglycemia associated with low body mass since weaning [16,17,18,19,20]. Notably, GK rats do not exhibit high plasma levels of free fatty acids, total cholesterol, LDL, HDL, and triglycerides up to 4 months of age [19,21]. Indeed, Tranæs et al. (2021) [22] reported that IR in the Asian population is independent of plasma lipid and lipoprotein levels, confirming previous findings [23].

Systemic inflammation has been reported in GK rats from the neonatal stage to 5 months of age [17,19]. The increased expression of genes induced by interferons (Ifit1 and Iigp1) is associated with chronic inflammation in the WAT and liver of these animals [19]. Additionally, Mongkolpathumrat et al. (2019) measured the gene expression (mRNA) of IL-6, TNF-α, and IL-1β in several organs and cytokine levels in the serum of four-month-old GK rats [24]. The authors reported increased IL-6 and IL-1β expression and production in the liver, IL-6 production in the kidney, IL-6 and TNF-α production and IL-1β expression in the brain, and elevated serum TNF-α levels. In addition, previous studies from our group demonstrated upregulated IL-1β concentrations in the duodenum and jejunum, and NF-κB p65 concentrations in the duodenum, jejunum, and ileum [25]. As part of the systemic inflammatory feature, macrophage infiltration has been reported in various tissues such as pancreatic islets, the kidneys, intestine, and retina by several research groups [10,26,27,28,29,30,31,32].

Macrophages are tissue-resident innate immune cells capable of reprogramming their metabolism to promote inflammation. The M2 (anti-inflammatory) type is the default programming macrophage. M2 macrophages exhibit high activities of oxidative phosphorylation, fatty acid oxidation pathways, and glutaminolysis. These cells play a key role in inflammation resolution and tissue repair by producing cell growth factors such as transforming growth factor beta (TGF-β) and anti-inflammatory cytokines such as IL-10 [33]. Polarization to the M1 type (pro-inflammatory) can occur by molecular patterns associated with pathogens (PAMPs) stimulus, such as lipopolysaccharides (LPS); molecular patterns associated with tissue damage (DAMPs); and interferon gamma (IFN-γ), secreted mainly by T helper 1 lymphocytes (Th-1) and TNF-α. M1 cells produce reactive oxygen (ROS) and nitrogen (RNS) species and pro-inflammatory cytokines, such as TNF-α, IL-1β, IL-6, IL-12 and IL-23. These macrophages display augmented glycolytic and pentose pathway activities [33,34,35,36].

In the present study, we hypothesized that macrophages from GK rats are in a permanent pro-inflammatory state and play a vital role in the systemic low-grade inflammation state that induces IR without obesity. Towards this goal, we measured the proportions of M1 and M2 macrophages from the peritoneal cavity of GK and control rats, the production of pro- and anti-inflammatory cytokines (such as IL-1α, TNF-α and IL-6) and stimulating and chemoattractant factors (CXCL-1, MCP-1 and GM-CSF) in the presence or absence of LPS, and ROS production in the presence and absence of PMA. The obtained results provide novel insights into the contribution of macrophages to the pro-inflammatory state of GK rats and their role in the onset and development of IR under non-obese conditions.

## 2. Results

### 2.1. Characterization of Non-Obese Diabetic Feature

**Figure 1:**
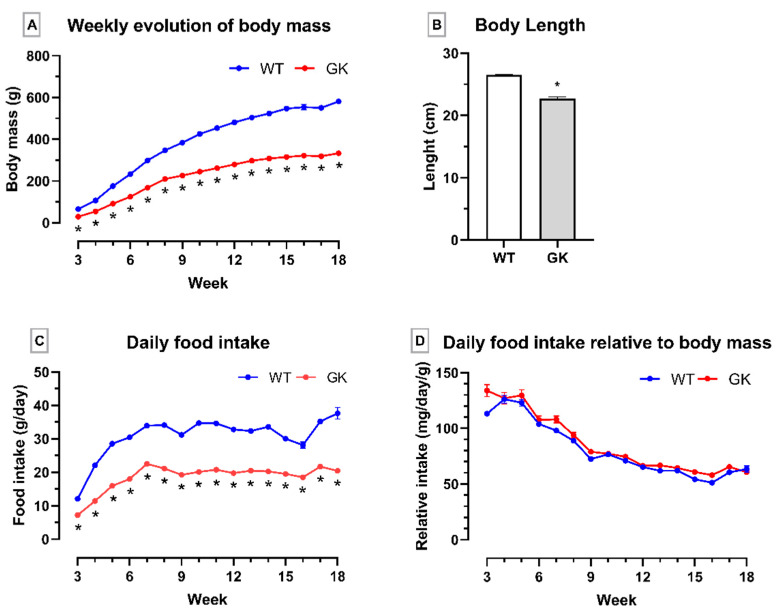
Body mass, food intake, and nasal–anal length of Goto-Kakizaki (GK) and Wistar (WT) rats. (**A**) Weekly evaluation of body mass, (**B**) nasal–anal length, (**C**) daily food intake, and (**D**) daily food intake relative to body mass. Results are presented as the mean ± SEM. The two groups were compared using one-way ANOVA (**A**,**C**,**D**) and Mann–Whitney’s test (**B**) for * *p* < 0.05. Number of animals used: WT = 7–9 and GK = 9. The individual numbers are in the Supplemental Data (Table S1).

The animals’ body mass and food intake were recorded weekly to confirm the non-obese diabetic state of GK rats in our colony. The nasal–anal length was measured at eighteen weeks of age on the day of euthanasia after the animals were anesthetized. The GK rats had a smaller body mass (Figure 1A) and were 14% shorter in length (Figure 1B). The GK rats also exhibited lower daily food intake (Figure 1C). However, when normalizing their food intake for the animal’s body mass, the values were similar (Figure 1D).

The animals’ blood glucose levels were measured at 21, 60, and 120 days of age (Table S3). The animals were fasted for 12 h, and the blood glucose levels were measured. We observed that fasting glycemia in GK rats is greater than in the WT group, indicating diabetes since weaning (Table 1).

### 2.2. Proportions of M1 and M2 Macrophage Phenotypes in the Peritoneal Cavity

**Figure 2:**
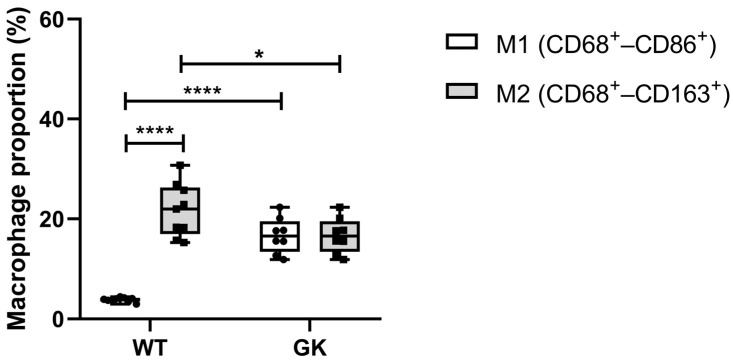
Percentage of M1 and M2 macrophages isolated from the peritoneal cavity of Wistar (WT) and Goto-Kakizaki (GK) rats. CD68^+^ = total macrophages; CD68^+^ CD86^+^ = M1 macrophages; CD68^+^ CD163^+^ = M2 macrophages. Two-way *ANOVA* and multiple comparisons by Sidak’s test were used to compare WT and GK rats (* *p* < 0.05; **** *p* < 0.0001). Number of animals: WT = 9 and GK = 8. Results are presented as the mean ± SEM. The individual numbers are described in the Supplemental Data (Table S4).

Peritoneal macrophages were obtained from eighteen-week-old rats, as described in the Materials and Methods. The representative dot plots from flow cytometry are presented in the Supplementary Material (Table S4). We considered CD68^+^ CD86^+^ cells M1 macrophages, and CD68^+^ CD163^+^ cells as M2 (Figure S1). The mean of cells positive for CD68 staining corresponds to 98.07% in WT and 97.56% in GK rats. The predominant type in the peritoneum of both groups of animals is M2 (Figure 2). However, GK rats have 60% more M1 and 23% less M2 macrophages compared to the Wistar control group.

### 2.3. Production of Reactive Oxygen Species by Isolated Macrophages

The obtained macrophages from eighteen-week-old rats were subjected to chemiluminescence analyses, as described in the Materials and Methods. This approach allowed us to evaluate each group’s response to the stimulus through the ratio between the means of the PMA/basal results that were significant. It was found that ROS production increased by 90 times in macrophages from GK rats and 36 times in those from Wistar control rats (Table 2), highlighting the effectiveness of the methodology and the differences in ROS production between the two rat strains.

### 2.4. Cytokine Concentration in Cultured Peritoneal Macrophage Supernatant

The assays were performed with the supernatant of macrophages isolated from the peritoneum cavity and cultured in the presence and absence of LPS (Table 3). GM-CSF, IFN-γ, IL-12p70, IL-17A and IL-1β were detected only in LPS-stimulated macrophages.

The GK LPS-stimulated macrophages produced 1.6 times more GM-CSF, 1.5 times more MCP-1 and 3.3 times more TNF-α compared to WT LPS-stimulated macrophages (Table 3). Additionally, there was a clear trend towards greater production of MCP-1, IL-1α and TNF-α under basal conditions in macrophages from GK animals compared with controls (Table 3).

## 3. Discussion

Our initial characterization of this model demonstrated that GK animals exhibited a lower body mass compared to the Wistar control group from weaning until eighteen weeks of age. This distinct feature has been previously linked to a smaller growth in length [20]. Our research group has confirmed this by observing a smaller nasal–anal length in GK rats, a finding also reported by others [18,25,37,38].

The GK rats exhibited an impaired response in the glucose tolerance test (GTT) and insulin tolerance test (ITT) at the ages of 21, 60, and 120 days, with elevated blood glucose levels compared to Wistar. At the age of 21 days, the GK rats also presented hyperinsulinemia, with increased plasma insulin levels than the control group. Our group published this data, and the same animals were used in the present study [39].

The normalized food intake by the animal’s body mass was similar between groups. Hyperphagia was reported in GK rats [40] and was attributed to lower appetite suppressing the activity of pro-opiomelanocortin (POMC) neurons impaired by hyperglycemia during the weaning period [41,42]. Herein, we reported high food intake by GK rats at weaning and around two months of age (seven weeks). Xue et al. (2011) and Kuwabara et al. (2017) reported higher food intake relative to body mass in GK rats from two months to eighteen weeks.

Throughout the entire experimental period (21 days to 18 weeks old), GK rats presented with a low body mass, which could be due to impaired differentiation of pre-adipocytes into mature adipocytes, making it difficult to store fat in the WAT [19]. Although we did not evaluate the fat deposits, Pereira et al. (2021) reported that GK rats have reduced epididymal, retroperitoneal, and mesenteric adipose tissue mass weight. Regarding glycemia, GK rats exhibited fasting hyperglycemia and glucose intolerance since weaning, which agrees with Portha et al. (2012) and Ando et al. (2018). This glucose intolerance is observed in 3- and 18-week-old GK rats, as reported by our group [18,25,37,38,43] and others [44,45,46].

The predominant macrophage phenotype in the peritoneum of Wistar and GK rats is M2. This result was expected since this is the standard programming of resident macrophages [33]. However, GK rats have more M1 and less M2 compared to Wistar controls, thus demonstrating macrophage signalization reprogramming. The increased production of GM-CSF, MPC-1 and TNF-α under stimuli further indicates this reprogramming towards a pro-inflammatory macrophage phenotype.

M1 and M2 are mainly based on the expression of CD86 (M1) and CD163 (M2). However, the association with intrinsic markers such as IL-4, IL-13, and IL-10 allows for more specific phenotyping, especially of the so-called M2-like macrophages [47,48].

M2-like macrophages represent a subset of M2 macrophages, including M2a, characterized by the expression of CD206 and CD209 and the secretion of IL-10, TGF-β, and IL-4; M2b, which expresses CD86 and produces TNF-α, IL-6, and IL-1; and M2c and M2d, both marked by the expression of CD163. The discovery of these subsets points out the ability of macrophages to transition between their polarization, expression, and function [48].

In this study, we used markers for M1 and M2 phenotypes. This experimental approach could be now designed to capture the full spectrum of macrophage diversity, as described above. Using additional markers and more refined methods could provide a more comprehensive understanding of macrophage subsets. Nonetheless, the M1/M2 classification remains a valuable framework for macrophage research in many contexts. 

GK rats have a high infiltration of macrophages in tissues or organs that modulate the microenvironment to an inflammatory state, such as pancreatic islets and the retina. Hachana et al. (2020) reported a subretinal infiltration of activated macrophages accompanied by gliosis reactivity, leading to an inflammatory process in the retinas of seven-month-old GK rats. This macrophage-related inflammatory condition has also been reported by Zhang et al. (2014) in the bone marrow-derived macrophages of GK rats, which presented higher levels of IRAK4, IkBa, p-p38 and HuR and higher production of IL-6 under LPS stimulation [49].

GK rats also present higher production and expression of MCP-1, as reported by Homo-Delarche et al. (2006) and Matafome et al. (2012) in 1- and 18-week-old rats, respectively [26,50]. Homo-Delarche reported a high expression of MCP-1 and TNF-α in pancreatic islets, alongside a macrophage infiltration, and Matafome et al. observed high levels of MCP-1 in the fibrotic and glycated regions of epididymal adipose tissue of GK rats, the same areas where macrophage infiltration was detected.

In addition, the greater production of GM-CSF, MCP-1 and TNF-α following LPS stimulation may be responsible for the higher M1 phenotype in GK rats, since these factors are known for their immunomodulatory capacities [51]. For example, the production of GM-CSF upregulates the expression of interferon regulatory factor 4 (IRF4), which supports macrophage polarization into a pro-inflammatory profile, and cytokines such as TNF-α, MCP-1 and IL-6 activate and assist in the proliferation of lymphoid cells during inflammatory responses [52,53,54].

Regarding ROS production, the basal response of GK rats was three times less than that of Wistar. After stimulation with PMA, the production was increased 90 times in the diabetic group. However, even with a response 3× higher than the control group, the ROS production is 33% less than that of Wistars. This decrease in peritoneum macrophage response is expected. Prior work shows that peritoneum cells of diabetic rats have decreased immune functions (e.g., cytokine release) [55].

Hyperglycemia also plays a direct role in macrophage polarization towards the M1 phenotype through the formation of advanced glycation end products that contribute to greater expression and production of pro-inflammatory cytokines (e.g., TNF-α) [56,57].

During intraperitoneal lavage for total cell isolation, some cells remain in the cavity, which is a limitation of the protocol. So, due to limitations in the macrophage isolation method, the total number of macrophages isolated from the peritoneal cavity by lavage is not precisely determined. This methodological constraint could impact the accuracy of quantifying the peritoneal macrophage population.

According to our previous search, CD206 antibodies for flow cytometry are not available for rats. This fact may significantly impact a comprehensive assessment of macrophage diversity, potentially restricting our ability to capture the full spectrum of macrophage phenotypes and the robustness of our findings.

Our results reveal that GK rat macrophages exhibit a pro-inflammatory profile, evidenced by the higher percentage of M1 and lower M2 macrophages, higher production of GM-CSF, MCP-1 and TNF-α upon stimulation, and a trend towards greater production of TNF-α, MCP-1, and IL-α under basal conditions compared to the control. Thus, these data suggest that macrophages play a central role in establishing the systemic inflammation that leads to IR and diabetes in GK rats.

**Figure 3:**
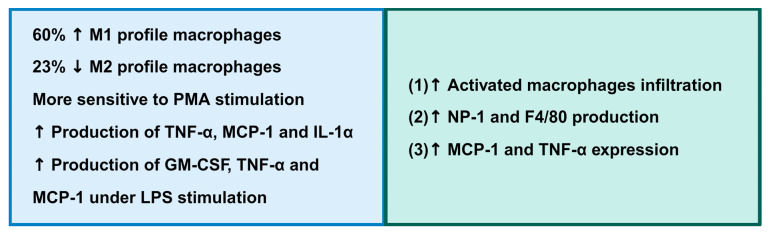
Indicators for the pro-inflammatory state of macrophage from Goto-Kakizaki rats, as described in this study and by others. ↑ = higher and ↓ = lower. Citations: (1—Omri et al., 2011 [31]); (2—Zhang; Sioud, 2023 [48]); (3—Pereira et al., 2021 [25]).

It is important to highlight that the literature has not provided information about the physiology of GK peritoneal macrophages until now, which reinforces the relevance of the results found in this study (Figure 3). In this sense, the present work contributes to a better understanding of the physiology of peritoneal macrophages from GK rats and the role of these cells in IR, providing valuable insights into measures for treating individuals with T2DM.

## 4. Materials and Methods

### 4.1. Animals

Male Wistar and Goto-Kakizaki (GK) rats obtained from the Charles River Laboratories International Inc. (Wilmington, MA, USA) were housed in the Interdisciplinary Post-graduate Program in Health Sciences animal facility at Cruzeiro do Sul University, São Paulo, Brazil. The temperature of the room was 23 ± 2 °C, 50 ± 10% humidity, with a light/dark cycle of 12 h. Age-matched animals had *ad libitum* access to water and standard rodent chow (Nuvilab^®^-Quimtia, Colombo, PR, Brazil). Weight and food intake were monitored from weaning (21 days) to 18 weeks of age. Nasal–anal length and macrophage assessments were recorded at 18 weeks of age. The Animal Ethical Committee at the Cruzeiro do Sul University (024-2017) approved the experimental procedures.

### 4.2. Body Mass and Food Intake Measurement

Three animals from the same group were kept per cage, totaling nine Wistar and nine GK rats. The body mass of each animal, the weight of the food offered, and the amount of food remaining were measured twice a week on an electronic scale, from weaning to euthanasia (21 days to 18 weeks of age). At the end of each week, the mean body mass and the standard error of the mean (SEM) for each group were calculated (in grams) to determine the weekly body mass.

The daily food intake per animal (g/day) was estimated by measuring the difference between the food weight offered and the remaining food in the cage. This value was then divided by 7 (seven days a week) and by 3 (three animals per cage) to provide a comprehensive average for each group. The daily food intake relative to body mass (mg/day/g) was calculated by the ratio between the weekly daily food intake and the body mass measured of each animal, with the results presented as mean and SEM (Table S1).

### 4.3. Nasal–Anal Length Measurement and Euthanasia

At 18 weeks of age, we anesthetized the rats with an intramuscular injection of ketamine (75 mg/kg) and xylazine (10 mg/kg) [58]. Nasal–anal length was measured in the anesthetized rats using a tape measure (Table S2), and euthanasia was performed by decapitation using a guillotine.

### 4.4. Macrophage Isolation from Peritoneal Washing

Macrophages were obtained by washing the peritoneal cavity without any prior stimulus, which would affect the cells’ metabolism, function, and phenotype [59].

We injected into the peritoneum 40 mL of sterile, ice-cold Roswell Park Memorial Institute (RPMI)-1640 culture medium supplemented with L-glutamine (2 mM), sodium bicarbonate (24 mM), HEPES (22 mM), fetal bovine serum (FBS; 10%), penicillin (100 IU/mL), and streptomycin (100 μg/mL) (Sigma-Aldrich, St. Louis, MI, USA). The peritoneum was massaged to displace cells attached to the serosa and peritoneal membranes into the culture medium, with the same number of massages (20 times) applied to all animals. We collected the peritoneal medium containing cells using a sterile Pasteur pipette. The cell suspension was centrifuged at 400× *g* for 10 min at 4 °C.

Following the centrifugation and discarding of the supernatant, the red blood cells were then lysed with 5 mL of a lysis solution [150 mM ammonium chloride, 10 mM sodium bicarbonate and 0.1 mM ethylenediaminetetraacetic acid (EDTA)]. After another centrifugation and discarding of the supernatant, the cells were resuspended in 1 mL of RPMI-1640 culture medium, and an aliquot was removed for cell counting and cell viability determinations [60,61].

### 4.5. Cell Viability Assay

An aliquot of cells from a 1 mL suspension of peritoneal lavage was diluted 50 times in an aqueous solution of 0.1% trypan blue (Sigma-Aldrich) and counted in a hemocytometer (Neubauer chamber) on a Nikon Eclipse TS100 inverted microscope (Minato, Tokyo, Japan). Blue-stained cells were considered dead [62]. We calculated the cellular yield in 1 mL and the percentage of viable cells.

### 4.6. Macrophage Culture

Macrophages were cultured (1 × $10^{6}$ peritoneal lavage cells/well) in 24-well plates, which were maintained in an incubator (Sanyo MCO-17AC, Osaka, Japan) at 37 °C and 5% of CO_2_ for 2 h. After this period, the culture medium was discarded. The adhered macrophages were washed with 500 μL sterile and heated phosphate-buffered saline (PBS, 137 mM sodium chloride, 2.7 mM potassium chloride, 8.1 mM anhydrous dibasic sodium phosphate, and 1.5 mM anhydrous monobasic potassium phosphate) to remove other cell types, such as B and T lymphocytes [61]. We added 1 mL RPMI-1640 medium with or without 500 ng/mL LPS (*Escherichia coli* O111:B4, Sigma-Aldrich) in each well, six wells per animal, three with and three without LPS stimuli. Cells were kept in culture for 6 h, and the supernatant was collected [63].

### 4.7. Cytokine Concentration in Cultured Macrophage Medium

The supernatant samples, collected as described above, were stored at −80 °C until the cytokine concentration was measured by flow cytometry using the LEGENDplex™ Rat Inflammation Panel (BioLegend^®^, San Diego, CA, USA). This assay allows the determination of 13 cytokines simultaneously, namely: IL-1β, IL-6, IL-10, IL-18, IL-1α, IL-12p70, IL-17A, IL-33, TNF-α, IFN-γ, ligand C-X-C family chemokine 1 or keratinocyte-derived chemokine (CXCL1/KC), C-C family chemokine 2 ligand or monocyte chemoattractant protein 1 (CCL2/MCP-1), and granulocyte-macrophage colony-stimulating factor (GM-CSF). A 1:4 serial curve was prepared with eight points using the kit’s standard reagent. Samples were diluted 4× in assay buffer. In a 96-well plate (V-shaped bottom), 12.5 μL LEGENDplex™ Matrix C were added to the wells previously defined for the curve preparation, and 12.5 μL assay buffer was added to the wells corresponding to the samples. The standards and samples were added to the corresponding wells, and 12.5 μL premixed beads reagent, previously vortexed, were added to all wells. The plate was sealed, wrapped in aluminum foil and stirred at approximately 800 rpm for 2 h at room temperature.

After 2 h of incubation, the plates were centrifuged at 250× *g* at room temperature, and the supernatant was removed using a multichannel pipette. For the washing, 100 μL washing buffer were added, and after 1 min of shaking, the plate was centrifuged and the supernatant removed as described above. The detection antibody (in 12.5 μL) was added, and the plate was sealed and shaken for 1 h at room temperature. After agitation, 12.5 μL LEGENDplex™ SA-PE were added, and the plate was sealed again and agitated for 30 min at room temperature. After this period, the plate was centrifuged and washed, and the beads were resuspended in 120 μL washing buffer. Data were acquired on a BD Accuri™ C6 Plus Flow Cytometer (BD Biosciences, San Jose, CA, USA) at slow flow; 4000 events were recorded per sample. The analysis of the data was performed using LEGENDplex™ online software version 8.

### 4.8. Proportion of M1 and M2 Macrophages

Cells from the peritoneal cavity washing were processed, and 2 × $10^{6}$ cells were centrifuged at 250× *g* for 10 min at room temperature and thoroughly resuspended with 1 mL of cold BD Cytofix™ Fixation Buffer (BD Biosciences, San Jose, CA, USA). Afterward, cells were washed twice at room temperature in a stain buffer (FBS) and centrifuged at 250× *g* for 10 min at room temperature. Cells were stored in 90% FBS/10% DMSO at −80 °C.

The proportion of M1 and M2 macrophages was determined by flow cytometry using the following monoclonal antibodies conjugated to fluorophores: for total macrophages, Alexa Fluor^®^ 647 anti-CD68 (1:20, Bio-Rad, Hercules, CA, USA); for M1, Phycoerythrin anti-CD86 (1:50, BD Biosciences); and for M2, Fluorescein isothiocyanate anti-CD163 (1:20, Bio-Rad) [64]. After defrosting, the cells were stained with anti-CD86 and anti-CD163 surface antibodies, permeabilized with Perm/Wash™ buffer (BD Biosciences), and incubated with the anti-CD68 intracellular antibody for 30 min at room temperature. Isotype controls for each fluorochrome were used. Twenty thousand events were acquired. Dot plots were analyzed using BD-Accuri software version 1.0.264.21 (BD Biosciences).

### 4.9. Production of Reactive Oxygen Species (ROS) by Cultured Peritoneal Macrophages

In a 96-well plate, 1 × $10^{6}$ cells were placed per well in PBS and treated with 1 mM luminol (Sigma-Aldrich) in the presence or absence of phorbol myristate acetate (PMA, 50 ng/mL, Sigma-Aldrich) for a final volume of 30 μL. The reading was performed on a FLUOstar^®^ Omega luminometer (BMG Labtech, Ortenberg, Baden-Württemberg, Germany) immediately after the PMA addition and monitored for 30 min [65].

### 4.10. Statistical Analysis

All statistical analyses were performed using GraphPad Prism 8.0.1 software. Before the analyses, the raw data were subjected to outlier identification (ROUT) and normality (Shapiro-Wilk) tests.

Student’s *t*-test was employed for comparing two groups, with Welch’s correction applied in cases where sample standard deviations were unequal. When data missed normality assumptions, the Mann-Whitney test was applied.

The Bonferroni post-hoc test was used following two-way ANOVA analysis, and post-hoc Tukey’s test was applied after performing repeated measures one-way ANOVA test. A significance level was defined as *p* < 0.05.

## Figures and Tables

**Table 1:** Blood glucose levels of Goto-Kakizaki (GK) and Wistar (WT) rats at weaning, eight and eighteen weeks of age.

Group	Age	Fasting Glucose
WT	Weaning	114.4 ± 4.2
	Eight weeks old	89.8 ± 1.6
	Eighteen weeks old	90.0 ± 3.4
GK	Weaning	137.1 ± 4.0 *
	Eight weeks old	157.6 ± 6.9 **
	Eighteen weeks old	130.6 ± 5.1 **

Comparisons between groups were performed using the Student’s *t*-test; * *p* < 0.05 and ** *p* < 0.0001. Number of animals: WT = 8 and GK = 9 (weaning and eighteen weeks); WT = 4 and GK = 5 (eight weeks). Results are presented as the mean ± SEM. The individual numbers are in the Supplemental Data (Table S3).

**Table 2:** Production of reactive oxygen species by macrophages from Wistar (WT) and Goto-Kakizaki (GK) rats over the course of 30 min with and without phorbol myristate acetate (PMA) stimulation.

Group	Basal (RU) *	PMA (RU) *	PMA/Basal Ratio
WT	3.6 ± 1.2	132.6 ± 32.7	36
GK	0.98 ± 0.18	88.3 ± 13.7	90

* Results are presented as the mean ± SEM. The PMA/Basal ratio indicates the response to PMA compared to the basal condition. Groups were compared using two-way ANOVA. RU = Relative Unit. Number of animals used: WT = 7 and GK = 8. The individual numbers are in the Supplemental Data (Table S5).

**Table 3:** Concentration of cytokines in the supernatant from peritoneal macrophages from Wistar (WT) and Goto-Kakizaki (GK) rats cultured for 6 h in basal or LPS-stimulated condition.

	WT		GK	
Cytokine	Mean Concentration ± SEM (pg/mL)	Number of Animals	Mean Concentration ± SEM (pg/mL)	Number of Animals
MCP-1	10,872 ± 2155	8	24,136 ± 13,772	5
IL-1α	9.92 ± 1.01	5	85.0 ± 39.2	6
TNF-α	115 ± 33.9	8	531 ± 208	7
IL-6	53.3 ± 6.12	8	48.1 ± 11.5	6
CXCL-1	2516 ± 504	8	4834 ± 1684	7
IL-18	67.6 ± 7.89	7	121 ± 38.9	7
GM-CSF *	176 ± 12.3	8	289 ± 33.8	5
TNF-α **	4610 ± 626	9	15,219 ± 2495	5
MCP-1 *	21,317 ± 2724	9	31,800 ± 4281	7
CXCL-1	10,916 ± 3811	9	7437 ± 4171	5
IFN-γ	102 ± 34.9	7	330 ± 125	6
IL-18	233 ± 69.6	7	244 ± 88.2	5
IL-6	52,125 ± 15,280	5	35,950 ± 12,211	6
IL-1α	704 ± 70.3	8	610 ± 139	6
IL-17A	8.01 ± 2.41	4	10.8 ± 1.94	5
IL-1β	20.3 ± 3.74	9	23.0 ± 4.9	6
IL-12p70	167 ± 59.3	4	143 ± 24.9	6

Monocyte chemoattractant protein 1 (MCP-1), interleukin-1 alpha (IL-1α), tumor necrosis factor-alpha (TNF-α), interleukin-6 (IL-6), C-X-C family chemokine 1 ligand (CXCL-1) and interleukin-18 (IL-18) detected in basal condition. Granulocyte-macrophage colony-stimulation factor (GM-CSF), tumor necrosis factor-alpha (TNF-α), monocyte chemoattractant protein 1 (MCP-1), C-X-C family chemokine 1 ligand (CXCL-1), interferon-gamma (IFN-γ), interleukin-18 (IL-18), interleukin-6 (IL-6), interleukin-1 alpha (IL-1α), interleukin-17A (IL-17A), interleukin-1 beta (IL-1β) and interleukin-12, p70 (IL-12p70) detected under lipopolysaccharide (LPS) stimuli (500 ng/mL). Student’s t-test or Mann–Whitney test was used for comparisons between the WT and GK rats (* *p* < 0.05 and ** *p* < 0.01). The individual numbers are in the Supplemental Data (Table S6).

## Data Availability

The data presented in this study are openly available in Zenodo at 10.5281/zenodo.12786331.

## Supplementary Materials

The following supporting information can be downloaded at: https://www.mdpi.com/article/10.3390/ijms251910240/s1.

## References

[1] IDF Diabetes Atlas 2021|IDF Diabetes Atlas. https://diabetesatlas.org/atlas/tenth-edition/.

[2] DeFronzo R.A., Ferrannini E., Groop L., Henry R.R., Herman W.H., Holst J.J., Hu F.B., Kahn C.R., Raz I., Shulman G.I. (2015). Type 2 Diabetes Mellitus. Nat. Rev. Dis. Primers.

[3] Hirabara S.M., Gorjão R., Vinolo M.A., Rodrigues A.C., Nachbar R.T., Curi R. (2012). Molecular Targets Related to Inflammation and Insulin Resistance and Potential Interventions. J. Biomed. Biotechnol..

[4] Kim J.K. (2012). Endothelial Nuclear Factor κB in Obesity and Aging: Is Endothelial Nuclear Factor κB a Master Regulator of Inflammation and Insulin Resistance?. Circulation.

[5] Rogero M.M., Calder P.C. (2018). Obesity, Inflammation, Toll-Like Receptor 4 and Fatty Acids. Nutrients.

[6] Kuryłowicz A., Koźniewski K. (2020). Anti-Inflammatory Strategies Targeting Metaflammation in Type 2 Diabetes. Molecules.

[7] Kwon H., Pessin J.E., Nillni E.A. (2018). Adipokines, Inflammation, and Insulin Resistance in Obesity. Textbook of Energy Balance, Neuropeptide Hormones, and Neuroendocrine Function.

[8] Olefsky J.M., Glass C.K. (2010). Macrophages, Inflammation, and Insulin Resistance. Annu. Rev. Physiol..

[9] Lauterbach M.A.R., Wunderlich F.T. (2017). Macrophage Function in Obesity-Induced Inflammation and Insulin Resistance. Pflug. Arch..

[10] Akash M.S.H., Rehman K., Chen S. (2013). Role of Inflammatory Mechanisms in Pathogenesis of Type 2 Diabetes Mellitus. J. Cell. Biochem..

[11] Chen J., Hendriks M., Chatzis A., Ramasamy S.K., Kusumbe A.P. (2020). Bone Vasculature and Bone Marrow Vascular Niches in Health and Disease. J. Bone Miner. Res..

[12] Brunetti P. (2007). The Lean Patient with Type 2 Diabetes: Characteristics and Therapy Challenge. Int. J. Clin. Pract..

[13] Kashima S., Inoue K., Matsumoto M., Akimoto K. (2015). Prevalence and Characteristics of Non-Obese Diabetes in Japanese Men and Women: The Yuport Medical Checkup Center Study. J. Diabetes.

[14] Chan J.C.N., Malik V., Jia W., Kadowaki T., Yajnik C.S., Yoon K.-H., Hu F.B. (2009). Diabetes in Asia: Epidemiology, Risk Factors, and Pathophysiology. JAMA.

[15] Goto Y., Kakizaki M., Masaki N. (1975). Spontaneous Diabetes Produced by Selective Breeding of Normal Wistar Rats. Proc. Jpn. Acad..

[16] Movassat J., Saulnier C., Serradas P., Portha B. (1997). Impaired Development of Pancreatic Beta-Cell Mass Is a Primary Event during the Progression to Diabetes in the GK Rat. Diabetologia.

[17] Portha B., Giroix M.-H., Tourrel-Cuzin C., Le-Stunff H., Movassat J. (2012). The GK Rat: A Prototype for the Study of Non-Overweight Type 2 Diabetes. Methods Mol. Biol..

[18] Kuwabara W.M.T., Panveloski-Costa A.C., Yokota C.N.F., Pereira J.N.B., Filho J.M., Torres R.P., Hirabara S.M., Curi R., Alba-Loureiro T.C. (2017). Comparison of Goto-Kakizaki Rats and High Fat Diet-Induced Obese Rats: Are They Reliable Models to Study Type 2 Diabetes Mellitus?. PLoS ONE.

[19] Xue B., Sukumaran S., Nie J., Jusko W.J., DuBois D.C., Almon R.R. (2011). Adipose Tissue Deficiency and Chronic Inflammation in Diabetic Goto-Kakizaki Rats. PLoS ONE.

[20] Movassat J., Bailbé D., Lubrano-Berthelier C., Picarel-Blanchot F., Bertin E., Mourot J., Portha B. (2008). Follow-up of GK Rats during Prediabetes Highlights Increased Insulin Action and Fat Deposition despite Low Insulin Secretion. Am. J. Physiol. Endocrinol. Metab..

[21] Wang X., DuBois D.C., Cao Y., Jusko W.J., Almon R.R. (2014). Diabetes Disease Progression in Goto-Kakizaki Rats: Effects of Salsalate Treatment. Diabetes Metab. Syndr. Obes..

[22] Tranæs K., Ding C., Chooi Y.C., Chan Z., Choo J., Leow M.K.-S., Magkos F. (2021). Dissociation Between Insulin Resistance and Abnormalities in Lipoprotein Particle Concentrations and Sizes in Normal-Weight Chinese Adults. Front. Nutr..

[23] Ding C., Chan Z., Chooi Y.C., Choo J., Sadananthan S.A., Chang A., Sasikala S., Michael N., Velan S.S., Magkos F. (2018). Regulation of Glucose Metabolism in Nondiabetic, Metabolically Obese Normal-Weight Asians. Am. J. Physiol. Endocrinol. Metab..

[24] Mongkolpathumrat P., Nokkaew N., Adulyaritthikul P., Kongpol K., Sanit J., Pankhong P., Kumphune S. (2019). Diabetes Induced Internal Organs Inflammation in Non-Obese Type 2 Diabetic Rats. J. App Pharm. Sci..

[25] Pereira J.N.B., Murata G.M., Sato F.T., Marosti A.R., de Oliveira Carvalho C.R., Curi R. (2021). Small Intestine Remodeling in Male Goto–Kakizaki Rats. Physiol. Rep..

[26] Homo-Delarche F., Calderari S., Irminger J.-C., Gangnerau M.-N., Coulaud J., Rickenbach K., Dolz M., Halban P., Portha B., Serradas P. (2006). Islet Inflammation and Fibrosis in a Spontaneous Model of Type 2 Diabetes, the GK Rat. Diabetes.

[27] Luo X., Pan L., Nie A., Wang Q., Gu Y., Li F., Zhang H., Li W., Li X. (2013). Liraglutide Protects Pancreatic Beta Cells during an Early Intervention in Gato-Kakizaki Rats. J. Diabetes.

[28] Calderari S., Irminger J.-C., Giroix M.-H., Ehses J.A., Gangnerau M.-N., Coulaud J., Rickenbach K., Gauguier D., Halban P., Serradas P. (2014). Regenerating 1 and 3b Gene Expression in the Pancreas of Type 2 Diabetic Goto-Kakizaki (GK) Rats. PLoS ONE.

[29] Cheng Z.J., Vaskonen T., Tikkanen I., Nurminen K., Ruskoaho H., Vapaatalo H., Muller D., Park J.K., Luft F.C., Mervaala E.M. (2001). Endothelial Dysfunction and Salt-Sensitive Hypertension in Spontaneously Diabetic Goto-Kakizaki Rats. Hypertension.

[30] Qiao Z., Wang X., Zhang H., Han J., Feng H., Wu Z. (2020). Single-Cell Transcriptomics Reveals That Metabolites Produced by Paenibacillus Bovis Sp. Nov. BD3526 Ameliorate Type 2 Diabetes in GK Rats by Downregulating the Inflammatory Response. Front. Microbiol..

[31] Omri S., Behar-Cohen F., de Kozak Y., Sennlaub F., Verissimo L.M., Jonet L., Savoldelli M., Omri B., Crisanti P. (2011). Microglia/Macrophages Migrate through Retinal Epithelium Barrier by a Transcellular Route in Diabetic Retinopathy: Role of PKCζ in the Goto Kakizaki Rat Model. Am. J. Pathol..

[32] Hachana S., Pouliot M., Couture R., Vaucher E. (2020). Diabetes-Induced Inflammation and Vascular Alterations in the Goto–Kakizaki Rat Retina. Curr. Eye Res..

[33] Viola A., Munari F., Sánchez-Rodríguez R., Scolaro T., Castegna A. (2019). The Metabolic Signature of Macrophage Responses. Front. Immunol..

[34] Italiani P., Töpfer E., Boraschi D., Boraschi D., Penton-Rol G. (2016). Chapter 7-Modulation of Macrophage Activation. Immune Rebalancing.

[35] Murray P.J. (2017). Macrophage Polarization. Annu. Rev. Physiol..

[36] Curi R., de Siqueira Mendes R., de Campos Crispin L.A., Norata G.D., Sampaio S.C., Newsholme P. (2017). A Past and Present Overview of Macrophage Metabolism and Functional Outcomes. Clin. Sci..

[37] Serdan T.D.A., Masi L.N., Pereira J.N.B., Rodrigues L.E., Alecrim A.L., Scervino M.V.M., Diniz V.L.S., Dos Santos A.A.C., Filho C.P.B.S., Alba-Loureiro T.C. (2021). Impaired Brown Adipose Tissue Is Differentially Modulated in Insulin-Resistant Obese Wistar and Type 2 Diabetic Goto-Kakizaki Rats. Biomed. Pharmacother..

[38] Borges J.C.O., Oliveira V.A.B., Serdan T.D.A., Silva F.L.R., Santos C.S., Pauferro J.R.B., Ribas A.S.F., Manoel R., Pereira A.C.G., Correa I.S. (2023). Brain Glucose Hypometabolism and Hippocampal Inflammation in Goto-Kakizaki Rats. Braz. J. Med. Biol. Res..

[39] Lobato T.B., Manoel R., Pereira A.C.G., Correa I.S., Iser-Bem P.N., Santos E.S.D.S., Pereira J.N.B., De Araújo M.J.L., Borges J.C.D.O., Pauferro J.R.B. (2024). Insulin Resistance in Nonobese Type 2 Diabetic Goto Kakizaki Rats Is Associated with a Proinflammatory T Lymphocyte Profile. FEBS Lett..

[40] Maekawa F., Fujiwara K., Kohno D., Kuramochi M., Kurita H., Yada T. (2006). Young Adult-Specific Hyperphagia in Diabetic Goto-Kakizaki Rats Is Associated with Leptin Resistance and Elevation of Neuropeptide Y mRNA in the Arcuate Nucleus. J. Neuroendocrinol..

[41] Ando A., Gantulga D., Nakata M., Maekawa F., Dezaki K., Ishibashi S., Yada T. (2018). Weaning Stage Hyperglycemia Induces Glucose-Insensitivity in Arcuate POMC Neurons and Hyperphagia in Type 2 Diabetic GK Rats. Neuropeptides.

[42] Toda C., Santoro A., Kim J.D., Diano S. (2017). POMC Neurons: From Birth to Death. Annu. Rev. Physiol..

[43] Panveloski-Costa A.C., Kuwabara W.M.T., Munhoz A.C., Lucena C.F., Curi R., Carpinelli A.R., Nunes M.T. (2020). The Insulin Resistance Is Reversed by Exogenous 3,5,3’triiodothyronine in Type 2 Diabetic Goto-Kakizaki Rats by an Inflammatory-Independent Pathway. Endocrine.

[44] Imai E., Shibata K. (2018). Oral Glucose Tolerance and Tryptophan Metabolism in Non-Obese and Non-Insulin-Dependent Diabetic Goto-Kakizaki Rats Fed High-Tryptophan Diets. J. Nutr. Sci. Vitaminol..

[45] Szkudelska K., Deniziak M., Hertig I., Wojciechowicz T., Tyczewska M., Jaroszewska M., Szkudelski T. (2019). Effects of Resveratrol in Goto-Kakizaki Rat, a Model of Type 2 Diabetes. Nutrients.

[46] Azul L., Leandro A., Boroumand P., Klip A., Seiça R., Sena C.M. (2020). Increased Inflammation, Oxidative Stress and a Reduction in Antioxidant Defense Enzymes in Perivascular Adipose Tissue Contribute to Vascular Dysfunction in Type 2 Diabetes. Free Radic. Biol. Med..

[47] Boutilier A.J., Elsawa S.F. (2021). Macrophage Polarization States in the Tumor Microenvironment. Int. J. Mol. Sci..

[48] Zhang Q., Sioud M. (2023). Tumor-Associated Macrophage Subsets: Shaping Polarization and Targeting. Int. J. Mol. Sci..

[49] Zhang X., Wang Y., Hu W., Li D., Zhou Z., Pan D., Wu W., Xu T. (2014). Interleukin-1/Toll-Like Receptor-Induced Nuclear Factor Kappa B Signaling Participates in Intima Hyperplasia after Carotid Artery Balloon Injury in Goto-Kakizaki Rats: A Potential Target Therapy Pathway. PLoS ONE.

[50] Matafome P., Santos-Silva D., Crisóstomo J., Rodrigues T., Rodrigues L., Sena C.M., Pereira P., Seiça R. (2012). Methylglyoxal Causes Structural and Functional Alterations in Adipose Tissue Independently of Obesity. Arch. Physiol. Biochem..

[51] Edgar L., Akbar N., Braithwaite A.T., Krausgruber T., Gallart-Ayala H., Bailey J., Corbin A.L., Khoyratty T.E., Chai J.T., Alkhalil M. (2021). Hyperglycemia Induces Trained Immunity in Macrophages and Their Precursors and Promotes Atherosclerosis. Circulation.

[52] Lee K.M.C., Achuthan A.A., Hamilton J.A. (2020). GM-CSF: A Promising Target in Inflammation and Autoimmunity. Immunotargets Ther..

[53] Koncz G., Jenei V., Tóth M., Váradi E., Kardos B., Bácsi A., Mázló A. (2023). Damage-Mediated Macrophage Polarization in Sterile Inflammation. Front. Immunol..

[54] Li H., Meng Y., He S., Tan X., Zhang Y., Zhang X., Wang L., Zheng W. (2022). Macrophages, Chronic Inflammation, and Insulin Resistance. Cells.

[55] Alba-Loureiro T.C., Pithon-Curi T.C., Curi R. (2008). Reduced Cytokine Production by Glycogen-Elicited Peritoneal Cells from Diabetic Rats. Shock.

[56] Luo M., Zhao F., Cheng H., Su M., Wang Y. (2024). Macrophage Polarization: An Important Role in Inflammatory Diseases. Front. Immunol..

[57] Wong A., Sun Q., Latif I.I., Karwi Q.G. (2024). Metabolic Flux in Macrophages in Obesity and Type-2 Diabetes. J. Pharm. Pharm. Sci..

[58] Neves S.M.P., Filho J.M., de Menezes E.W. (2016). Manual de Cuidados e Procedimentos com Animais de Laboratório do Biotério de Produção e Experimentação da FCF-IQ/USP.

[59] Pavlou S., Wang L., Xu H., Chen M. (2017). Higher Phagocytic Activity of Thioglycollate-Elicited Peritoneal Macrophages Is Related to Metabolic Status of the Cells. J. Inflamm..

[60] Zhang X., Goncalves R., Mosser D.M. (2008). The Isolation and Characterization of Murine Macrophages. Curr. Protoc. Immunol..

[61] Magdalon J., Vinolo M.A.R., Rodrigues H.G., Paschoal V.A., Torres R.P., Mancini-Filho J., Calder P.C., Hatanaka E., Curi R. (2012). Oral Administration of Oleic or Linoleic Acids Modulates the Production of Inflammatory Mediators by Rat Macrophages. Lipids.

[62] Johnson S., Nguyen V., Coder D. (2013). Assessment of Cell Viability. Curr. Protoc. Cytom..

[63] Mosser D.M., Zhang X. (2008). Activation of Murine Macrophages. Curr. Protoc. Immunol..

[64] Liu R.-H., Wen Y., Sun H.-Y., Liu C.-Y., Zhang Y.-F., Yang Y., Huang Q.-L., Tang J.-J., Huang C.-C., Tang L.-J. (2018). Abdominal Paracentesis Drainage Ameliorates Severe Acute Pancreatitis in Rats by Regulating the Polarization of Peritoneal Macrophages. World J. Gastroenterol..

[65] Hatanaka E., Levada-Pires A.C., Pithon-Curi T.C., Curi R. (2006). Systematic Study on ROS Production Induced by Oleic, Linoleic, and γ-Linolenic Acids in Human and Rat Neutrophils. Free. Radic. Biol. Med..

