# Supplementary material for: Evidence for a Pro-Inflammatory State of Macrophages from Non-Obese Type-2 Diabetic Goto-Kakizaki Rats
Source: Int J Mol Sci. 2024 Sep 24;25(19):10240. doi: 10.3390/ijms251910240 (PMC11477416; doi:10.3390/ijms251910240)

Figure S1. Flow cytometry analysis of peritoneal cells of (**A**) CD68+ CD86+ and (**B**) CD68+ CD163+ cells of Goto-Kakizaki (GK) rats, (**C**) CD68+ CD86+ and (**D**) CD68+ CD163+ cellsof Wistar (WT) rats and (**E**) negative control of CD68-CD86- cells and (**F**) negative control of CD68-CD163- cells.

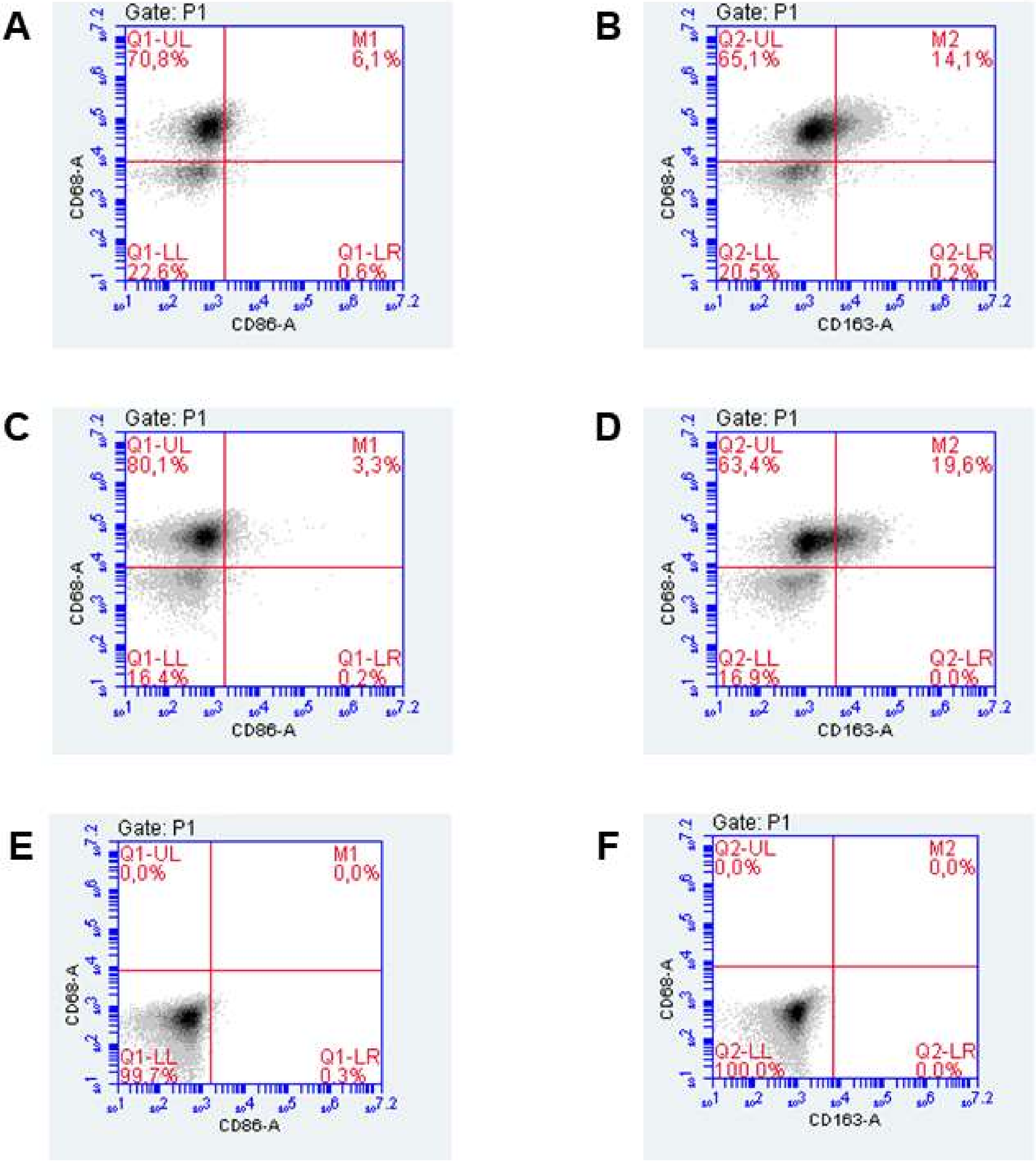

Supplement: Supplementary file 1 [file ijms-25-10240-s001.zip › Figure S1.pdf]
