# Supplementary material for: Evidence for a Pro-Inflammatory State of Macrophages from Non-Obese Type-2 Diabetic Goto-Kakizaki Rats
Source: Int J Mol Sci. 2024 Sep 24;25(19):10240. doi: 10.3390/ijms251910240 (PMC11477416; doi:10.3390/ijms251910240)
Supplement: Supplementary file 1 [file ijms-25-10240-s001.zip › Table S1.pdf]

Table S1. Weekly monitoring of average body mass and food consumption of Wistar and Goto-Kakizaki rats. SEM = Standard error of the mean; WT = Wistar; GK = Goto-Kakizaki. Number of animals: WT = 7–9 and GK = 9.

	WT						GK					
Age (weeks)	Body mass (g)	SEM (g)	Daily intake per animal in one week (g/day)	SEM (g/day)	Daily intake relative to body mass (mg/day/g)	SEM (mg/day/g)	Body mass (g)	SEM (g)	Daily intake per animal in one week (g/day)	SEM (g/day)	Daily intake relative to body mass (mg/day/g)	SEM (mg/day/g)
3	65.47	3.16	12.07	0.48	113.07	2.45	29.36	2.04	7.22	0.19	133.95	5.35
4	107.04	4.59	22.07	0.38	122.91	1.57	54.60	2.64	11.43	0.13	127.00	5.06
5	176.09	6.23	28.56	0.29	120.42	1.56	91.18	3.96	15.94	0.09	129.62	5.13
6	233.20	5.70	30.43	0.18	102.09	1.02	124.60	5.31	18.00	0.09	107.63	3.51
7	298.75	2.37	33.90	0.26	97.96	1.78	168.78	5.89	22.51	0.36	107.93	3.18
8	351.28	2.92	34.10	0.10	88.85	1.30	209.96	6.80	21.13	0.26	93.85	2.47
9	388.85	3.25	31.17	0.36	72.44	1.41	226.44	6.58	19.24	0.19	79.00	1.95
10	423.03	1.46	34.68	0.33	76.56	1.61	244.78	6.46	20.13	0.10	77.09	1.64
11	460.10	3.74	34.60	0.19	71.56	0.38	262.16	5.95	20.77	0.24	74.48	1.59
12	486.75	3.90	32.79	0.11	65.17	0.95	279.84	6.71	19.73	0.30	66.44	1.34
13	503.87	6.16	32.34	0.30	61.86	1.05	297.76	6.42	20.48	0.25	66.76	1.24
14	523.49	5.18	33.60	0.28	62.30	0.18	307.49	6.53	20.24	0.26	64.33	1.33
15	547.16	5.51	30.04	0.70	54.28	0.46	315.42	5.99	19.51	0.29	60.83	1.42
16	553.69	13.10	28.13	0.91	51.14	1.78	321.60	5.49	18.48	0.48	58.05	1.39
17	550.58	6.08	35.15	0.29	60.54	0.60	318.62	5.48	21.69	0.39	65.33	1.78
18	580.91	7.08	37.59	1.77	63.45	2.87	332.96	6.24	20.44	0.67	60.65	2.36
