# Supplementary material for: Evidence for a Pro-Inflammatory State of Macrophages from Non-Obese Type-2 Diabetic Goto-Kakizaki Rats
Source: Int J Mol Sci. 2024 Sep 24;25(19):10240. doi: 10.3390/ijms251910240 (PMC11477416; doi:10.3390/ijms251910240)
Supplement: Supplementary file 1 [file ijms-25-10240-s001.zip › Table S2.pdf]

Table S2. Nasal–anal length (cm) of eighteen weeks old Wistar and Goto-Kakizaki rats. SEM = Standard error of the mean. WT = Wistar; GK = Goto-Kakizaki. N=9.

	WT	GK
Animal number	Nasal–anal length (cm)	Nasal–anal length (cm)
1	26.5	23.5
2	26.7	23.5
3	26.5	23.0
4	26.5	22.0
5	26.6	21.3
6	25.5	22.0
7	26.0	23.0
8	26.5	23.0
9	26.5	23.0
Mean (g)	26.37	22.70
Standard error of the mean (SEM) (g)	0.13	0.25
