# Supplementary material for: Evidence for a Pro-Inflammatory State of Macrophages from Non-Obese Type-2 Diabetic Goto-Kakizaki Rats
Source: Int J Mol Sci. 2024 Sep 24;25(19):10240. doi: 10.3390/ijms251910240 (PMC11477416; doi:10.3390/ijms251910240)
Supplement: Supplementary file 1 [file ijms-25-10240-s001.zip › Table S3.pdf]

Table S3. Fasting blood glucose levels (mg/dL) of Wistar and Goto-Kakizaki rats. WT = Wistar; GK = Goto-Kakizaki. SEM = Standard error of the mean. Number of animals: WT = 8 and GK = 9 (weaning and eighteen weeks old); WT = 4 and GK = 5 (eight weeks).

	WT			GK		
	Fasting Glucose (mg/dL)					
Animal number	Weaning	Eight weeks old	Eighteen weeks old	Weaning	Eight weeks old	Eighteen weeks old
1	113	86	84	146	165	125
2	112	92	96	140	138	151
3	123	88	99	135	178	145
4	114	93	89	145	159	132
5	102		105	159	148	117
6	117		80	133		139
7	136		81	124		121
8	98		77	133		142
9				119		103
Mean (mg/dL)	114.38	89.75	88.88	137.11	157.60	130.56
Standard error of the mean (SEM) (mg/dL)	4.18	1.65	10.13	4.03	6.89	5.13
