# Supplementary material for: Evidence for a Pro-Inflammatory State of Macrophages from Non-Obese Type-2 Diabetic Goto-Kakizaki Rats
Source: Int J Mol Sci. 2024 Sep 24;25(19):10240. doi: 10.3390/ijms251910240 (PMC11477416; doi:10.3390/ijms251910240)
Supplement: Supplementary file 1 [file ijms-25-10240-s001.zip › Table S4.pdf]

Table S4. Percentage of M1 and M2 macrophages in the peritoneal cavity in Wistar and Goto-Kakizaki rats. WT = Wistar; GK = Goto-Kakizaki. SEM = Standard error of the mean. Number of animals: WT = 9 and GK = 8.

	WT		GK	
	Macrophage profile (%)			
Animal number	M1	M2	M1	M2
1	4.40	30.72	8.47	15.56
2	4.09	26.88	6.54	17.61
3	3.90	22.90	6.34	20.14
4	4.20	18.26	6.93	22.32
5	3.42	25.72	5.91	15.53
6	3.73	21.97	4.75	17.74
7	4.00	15.26	5.00	12.70
8	3.70	18.32	5.15	11.88
9	2.98	15.72		
Mean (%)	3.82	21.75	6.14	16.69
Standard error of the mean (SEM) (%)	0.0014	0.0180	0.0040	0.0120
