# Supplementary material for: Evidence for a Pro-Inflammatory State of Macrophages from Non-Obese Type-2 Diabetic Goto-Kakizaki Rats
Source: Int J Mol Sci. 2024 Sep 24;25(19):10240. doi: 10.3390/ijms251910240 (PMC11477416; doi:10.3390/ijms251910240)
Supplement: Supplementary file 1 [file ijms-25-10240-s001.zip › Table S5.pdf]

Table S5. Production of reactive oxygen species (ROS) by macrophages during 30 minutes with and without phorbol myristate acetate (PMA) stimulation in Wistar and Goto-Kakizaki rats. RU = Relative Unit. WT = Wistar; GK = Goto-Kakizaki. SEM = Standard error of the mean. Number of animals: WT = 7 and GK = 8.

	WT		GK	
	ROS production (RU)			
Animal number	Basal	PMA	Basal	PMA
1	1.23	40.09	1.11	111.11
2	1.19	105.70	1.03	88.81
3	0.54	51.92	0.73	65.00
4	4.36	150.72	0.77	107.35
5	3.15	108.84	0.72	42.04
6	9.81	175.93	1.91	120.38
7	5.22	295.16	0.22	31.81
8			1.39	140.01
Mean (RU)	3.64	132.62	0.98	88.31
Standard error of the mean (SEM) (RU)	1.22	32.73	0.18	13.67
