# Supplementary material for: Evidence for a Pro-Inflammatory State of Macrophages from Non-Obese Type-2 Diabetic Goto-Kakizaki Rats
Source: Int J Mol Sci. 2024 Sep 24;25(19):10240. doi: 10.3390/ijms251910240 (PMC11477416; doi:10.3390/ijms251910240)
Supplement: Supplementary file 1 [file ijms-25-10240-s001.zip › Table S6.pdf]

Table S6. Concentration of cytokines in the supernatant from peritoneal macrophages from Wistar (WT) and Goto-Kakizaki (GK) rats cultured for 6 hours in basal or LPS-stimulated condition

		Cytokines																
		Basal conditions (pg/mL)						LPS Stimulated (pg/mL)										
	Animal number	MCP-1	IL-1α	TNF-α	IL-6	CXCL-1	IL-18	GM-CSF	TNF-α	MCP-1	CXCL-1	IFN-γ	IL-18	IL-6	IL-1α	IL-17A	IL-1β	IL-12p70
WT	1	8,550	8.07	12.6	26.8	472	109	190	6,581	30,207	17,189	154	100	32,337	850	14.9	12.7	171
	2	14,469	12.0	70.1	47.5	1,904	55.5	150	4,238	21,821	15,385	32.5	89.4	109,556	498	4.56	4.12	331
	3	5,095	7.36	264	61.6	3,703	47.6	225	216	30,223	278	278	619	31,827	668	7.73	6.56	60.7
	4	9,408	9.82	32.6	35.0	847	73.1	150	5,869	12,519	498	73.0	205	57,895	602	4.84	30.8	104
	5	8,555	12.4	81.7	56.0	2,112	49.9	206	5,116	17,495	88.0	30.0	245	29,009	610		28.3	
	6	5,263		161	57.2	3,367	67.5	200	3,482	14,683	11,851	122	113		510		28.9	
	7	11,784		242	83.3	4,552	70.8	164	5,538	11,251	20,506	21.9	257		1,084		22.3	
	8	23,852		56.1	59.0	3,167		122	4,978	20,604	440				805		14.2	
	9								5,476	33,054	32,006						34.8	
Mean (pg/mL)		10,872	9.93	115	53.3	2,516	67.6	176	4,610	21,317	10,916	102	233	52,125	704	8.01	20.3	167
Standard error of the mean (SEM) (pg/mL)		2,155	1.01	33.9	6.12	504	7.89	12.3	626	2,724	3,811	34.9	69.6	15,280	70.3	2.41	3.74	59.3
GK	1	4,436	243	21.1	18.6	499	84.9	285	19,967	26,962	1,178	175	574	25,993	1,232	16.0	38.1	188
	2	28,725	10.8	1,504	20.0	10,613	167	211	14,396	39,232	13,914	467	280	13,880	391	14.3	37.8	158
	3	4,866	58.0	83.0	69.6	668	90.3	395	18,059	28,498	1,011	879	109	85,581	767	9.74	15.4	36.4
	4	76,123	20.6	840	44.8	6,215	28.7	227	17,767	51,644	20,696	32.3	117	8,049	504	8.65	13.5	108
	5	6,529	15.9	175	44.6	1,932	117	330	5,908	25,129	388	152	139	23,893	314	5.31	12.0	168
	6		161	234	90.9	3,07	30.0			16,51		276		58,303	455		21.4	200
	7			857		10,839	327			34,625								
Mean (pg/mL)		24,136	85.0	531	48.1	4,834	121	290	15,219	31,800	7,437	330	244	35,950	610	10.8	23.0	143
Standard error of the mean (SEM) (pg/mL)		13,772	39.1	208	11.5	1,684	38.9	33.8	2,495	4,281	4,171	125	88.2	12,211	139	1.94	4.90	24.9
